# International medical graduates: from brain drain to potential gain

**DOI:** 10.5116/ijme.5869.008a

**Published:** 2017-02-04

**Authors:** Muhammad H. Majeed, Ali Ahsan Ali, Fahad Saeed

**Affiliations:** 1 Department of Psychiatry, Natchaug Hospital, Norwich, CT, USA; 2 Department of Psychiatry, Brown University, Providence, RI, USA; 3 Department of Medicine, Divisions of Nephrology and Palliative Care, University of Rochester Medical Center, Rochester, NY, USA

**Keywords:** International, medical graduates, brain drain, potential gain

## Introduction

The international mobility of healthcare professionals is a well-documented phenomenon that has dynamic effects on global healthcare, labor markets, and the world economy. One aspect of this phenomenon—the movement of international medical graduates (IMGs) to the United States for residency training - is often criticized because of the “brain drain” from their home countries. We propose a different way of looking at this brain drain phenomenon and highlight the beneficial aspects of international physicians obtaining training in the United States and the potential impact such physicians could have on the healthcare systems of their own countries and possibly of the entire world.

### Immigration of professionals is a blessing in disguise

Currently, one in four physicians practicing in the United States is an IMG.^1^ In recent years, there has been a steady increase in the number of IMGs obtaining medical training positions in residency programs in the United States. In 2015, 3,641 non-US citizen IMGs obtained residency training positions^2^ as compared to 2010 when 2,881 secured such positions.^3^ National Residents Matching Program (NRMP) results continue to show that there is a consistent shortage of American medical graduates to fill all the residency positions available annually in the United States.^2^^,^^3^ These results challenge earlier projections that the country would not need any more IMGs by the year 2015.^4^ Moreover, the shortage of doctors in the United States is likely to continue to encourage IMGs to come to this country in the future. We believe that, with planning, these professionals could be the basis of a ‘brain circulation’ system that would help develop graduate medical education, healthcare infrastructure, and medical research in their home countries, which, in turn, would help to retain and repatriate the next generation of physicians.

### Careers on the move

Medical professionals from developing countries are moving across the globe and crossing geopolitical boundaries to find better professional, financial, and social conditions. India, Pakistan, Iran, China and Nigeria top the list of countries from which these aspiring physicians are coming. Obtaining a residency training position in the United States remains the top priority for many professionals because of the exceptional educational and research opportunities available for career development.^5^ In a world that is rapidly shifting towards globalized labor markets, super-specializations, and cutting-edge research paradigms, it would be counterproductive to discourage international physicians from seeking better medical training in the United States or in other countries that provide similar medical training opportunities.

### Brain drain can be useful for the home country

IMGs have the potential to impact the healthcare systems of their home countries while remaining in the United States and by returning periodically to their home countries. In the short term, even while in the United States, these physicians can contribute services via telemedicine, through educational webinars, or as visiting faculty members. They can also contribute by serving in emergency relief camps after natural disasters, by assisting efforts to upgrade hospitals and medical schools, and by fostering research and development in developing countries. In the long run, the participation of IMGs in the activities above can help bridge the gap in healthcare and medical education between developed and developing nations. In addition, the establishment of new, state-of-the-art medical centers and the improvement of established institutions in their own countries will require well-trained physicians and may encourage many IMGs to return home where they could be a significant resource in educating the next generation of physicians.

### Contributions towards services, academics, research and development

There are many ways that these physicians can provide services to their home countries. The following are examples of potential approaches, some of which are already in use.

From the pool of thousands of international physicians in the United States, interested physicians can be tapped to collaborate and provide various services via telemedicine. This would enable patients across the globe to obtain medical advice from highly trained physicians working abroad who also understand the local culture.

IMGs can help improve existing training programs and develop new specialty residency and fellowship training opportunities in developing countries by using the Internet or through on-site visits. For example, in the developing world, many medical training institutes lack opportunities for specialty training in such disciplines as child and adolescent psychiatry, bone marrow transplants, and palliative care.  With the help of international physicians trained in these specialties, the alumni of different medical schools can arrange lectures on sub-specialty subjects for medical students, residents and fellows across the globe. International physicians can teach these subjects from a distance via regular webinars or as visiting faculty members and by helping to develop specialty centers to train local doctors. They can also collaborate with local training programs to improve curriculum with local adjustments according to the needs of a particular country. These measures can provide opportunities for top quality professional training for the next generation of medical graduates.

Many IMGs decide not to remain permanently in the United States. For example, IMGs trained in the United States are already providing clinical services in many hospitals in Asia and Africa. It is not uncommon to see American-trained international physicians practicing across the globe in teaching hospitals that operate according to international standards and protocols, some of which are even run by United States institutions. Two such examples are the Cleveland Clinic in Abu Dhabi^6^ and Weil Cornell Medical College in Qatar.^7^

Another area where returning IMGs could be of benefit to their home countries is in medical tourism. Medical tourism is a growing business that attracts many American and European patients to developing countries because of the affordable cost of treatment, personalized care, short waiting time, and confidentiality.^8^ Most of these medical facilities are run by Western-trained physicians to ensure the same standard of care as in the United States. These growing commercial businesses are creating opportunities for American-trained physicians in developing countries and helping to reverse migration patterns.

International migration of health care professionals is dynamic and complex. This migration creates concern that such a brain drain might lead to a dwindling number of doctors per thousand population ratio in developing countries.^9^ Rather than adopting policies that would discourage international graduates from seeking training in the United States, it would be preferable to encourage international physicians to obtain such training and recognize the many benefits this could bring to their home countries and the global healthcare system.

## Conclusions

Highly skilled international physicians are valuable and recoverable assets for their countries of origin. We support the international movement of physicians because of the potential benefits of brain circulation. Irrespective of their location on the globe the potential for international physicians to contribute to the healthcare systems of the developing world is immense, although not yet fully appreciated.

### Conflicts of Interest

The authors declare that they have no conflict of interest.
